# TREM2 in Alzheimer’s Disease: Microglial Survival and Energy Metabolism

**DOI:** 10.3389/fnagi.2018.00395

**Published:** 2018-11-23

**Authors:** Honghua Zheng, Baoying Cheng, Yanfang Li, Xin Li, Xiaofen Chen, Yun-wu Zhang

**Affiliations:** ^1^Fujian Provincial Key Laboratory of Neurodegenerative Disease and Aging Research, Institute of Neuroscience, Medical College, Xiamen University, Xiamen, China; ^2^Shenzhen Research Institute, Xiamen University, Shenzhen, China

**Keywords:** Alzheimer’s disease, TREM2, microglia, survival, metabolism

## Abstract

Alzheimer’s disease (AD) is the leading cause of age-related dementia among the elderly population. Recent genetic studies have identified rare variants of the gene encoding the triggering receptor expressed on myeloid cells-2 (TREM2) as significant genetic risk factors in late-onset AD (LOAD). TREM2 is specifically expressed in brain microglia and modulates microglial functions in response to key AD pathologies such as amyloid-β (Aβ) plaques and tau tangles. In this review article, we discuss recent research progress in our understanding on the role of TREM2 in microglia and its relevance to AD pathologies. In addition, we discuss evidence describing new TREM2 ligands and the role of TREM2 signaling in microglial survival and energy metabolism. A comprehensive understanding of TREM2 function in the pathogenesis of AD offers a unique opportunity to explore the potential of this microglial receptor as an alternative target in AD therapy.

Alzheimer’s disease (AD), the most common form of dementia, is a neurodegenerative disease clinically characterized by progressive memory loss and cognitive deficits that are accompanied by pathological deposition of senile plaques and neurofibrillary tangles (NFTs) in the brain (Duyckaerts et al., 2009; Jankowsky and Zheng, 2017). AD is an age-related disease with a higher incidence in people over 65 years; after which AD incidence rate doubles every 5 years. By 2050, one new case of AD is expected to develop every 33 s, resulting in nearly 1 million new cases per year and a total of 115 million cases (Alzheimer’s Association, 2016). Given the heavy economic and social burdens associated with the care of AD patients, significant efforts, both public and private, have been undertaken to understand the pathogenesis of AD and develop early diagnostic methods and effective intervention therapies.

Microglia, known as resident immune cells in the brain, are classified based on morphology and functional state into resting (ramified) and activated/phagocytic (ameboid) microglia (Sarlus and Heneka, 2017). Fate-mapping studies have identified the immature yolk sac as the predominant source of microglia progenitors (Ginhoux et al., 2010; Schafer and Stevens, 2015). Recent advances in genomic research have uncovered significant contributions of microglia to inheritable disease risks for AD (Malik et al., 2015; Efthymiou and Goate, 2017; Salter and Stevens, 2017; Sarlus and Heneka, 2017). Genetic variants of the triggering receptor expressed on myeloid cells-2 (TREM2), which is dominantly expressed in microglia in central nervous system (CNS), result in increased risk of developing late-onset AD (LOAD) and other neurodegenerative disorders (Guerreiro et al., 2013; Jonsson et al., 2013; Painter et al., 2015; Ulrich and Holtzman, 2016). These genetic findings have attracted attention to the role of microglia in neurodegenerative diseases and TREM2 in regulating microglial function (Calcagno et al., 2018). Besides its neuroinflammatory and phagocytic role in pathogenesis, microglia plays important roles in clustering or limiting the diffusion or growth of amyloid-β (Aβ) plaques (Wang et al., 2016; Zhao R. et al., 2017) and tau tangles (Kitazawa et al., 2004; Bloom, 2014; Hung et al., 2016). Given our rapidly expanding understanding of the microglia function and dysfunction in CNS disorders, as well as the important roles of TREM2 in AD, microglia is generally accepted as playing a pivotal role in the maintenance of brain homeostasis (Salter and Stevens, 2017). In this review article, we discuss recent progress in our understanding of the role of TREM2 in microglia functions related to AD pathology, as well as signaling pathways activated by TREM2 ligands and their role in microglial survival and metabolism. Comprehensive understanding of TREM2 function in the pathogenesis of AD offers a unique opportunity to explore this microglial receptor as a target alternative to Aβ and tau in AD therapy.

## Neuropathological Hallmarks of AD

AD is pathologically characterized by a buildup of extracellular senile plaques composed of Aβ peptide, intracellular NFTs composed of hyperphosphorylated tau (p-tau) protein and reactive gliosis, including microgliosis (Efthymiou and Goate, 2017; Leyns and Holtzman, 2017). Currently, soluble β-amyloid precursor protein (APP), Aβ, tau and p-tau in cerebrospinal fluid and blood are candidate biomarkers for AD (Hampel et al., 2010; Shekhar et al., 2016; Lucey et al., 2017; Tatebe et al., 2017).

### Aβ Plaques

Aβ peptides, the main component of extracellular senile plaques, are sequentially cleaved from APP by distinct β- and γ-secretases (Zhang et al., 2014). In contrast, α-secretase cleavage of APP within the Aβ domain precludes the formation of toxic Aβ peptides (Nathalie and Jean-Noël, 2008). Aβ clearance involves phagocytosis and endocytosis via microglial scavenger receptors and extracellular degradation by Aβ-degrading enzymes (Hickman et al., 2018). Aβ can form oligomers, which are widely regarded as the most toxic and pathogenic forms of Aβ (Tu et al., 2014; Arbel-Ornath et al., 2017; Cline et al., 2018). It has been proposed that the over-production and/or decreased degradation of Aβ and subsequent formation of Aβ oligomers instigates the activation of microglia and astrocytes, dystrophy of neurites, and ultimately the symptoms of dementia (Viola and Klein, 2015; Brody et al., 2017; Cline et al., 2018; Doig, 2018). Microglia surround and limit Aβ plaques in AD brains and recent studies have reported reduced microglia clearance of Aβ in the absence of microglial scavenger receptors (Salter and Stevens, 2017; Ulland and Colonna, 2018). These findings support a direct link between abnormal microglial function and Aβ plaques in AD.

### Pathological Tau

Hyperphosphorylation of microtubule-associated protein tau (MAPT) is another key pathological hallmark of AD (Spillantini and Goedert, 1998; Lee et al., 2001; Wu et al., 2017). Tau is an axon-enriched protein that binds to and stabilizes microtubules, playing a crucial role in neuronal function. Studies have also revealed profound tau pathology in the visual system, leading to early retinal ganglion cell (RGC) damage in a mouse model of AD (Chiasseu et al., 2017). Recent studies propose inhibition or reduction of tau hyperphosphorylation as a potential therapeutic strategy for AD (Brunden et al., 2009; Li and Götz, 2017). Given that pathological tau correlates better with the degree of dementia than Aβ deposition, therapeutic strategies targeting pathological tau may provide a more promising approach for the treatment of AD. Because microglia can engulf, degrade and clear tau, it has recently been proposed microglia may play a significant role in the spreading of tau pathology (Leyns and Holtzman, 2017; Wang et al., 2017; Hickman et al., 2018). Nevertheless, the underlying pathways and the potential significance of microglia-mediated tau pathology require further investigation.

### Microgliopathy

Reactive gliosis, microglia dysfunction and neuroinflammation, sometimes described under the term “microgliopathy,” are pathological hallmarks of AD (Zhang et al., 2013; Sasaki, 2017) and other neurodegenerative diseases, including Parkinson’s disease (PD), amyotrophic lateral sclerosis (ALS) and frontal temporal dementia (FTD; Salter and Stevens, 2017). Molecules associated with these “microgliopathies” mainly include the 12 kDa DNAX activating protein (DAP12) and TREM2 in Nasu-Hakola disease (NHD; Paloneva et al., 2000, 2002); and the colony stimulating factor 1 receptor (CSF1R) in hereditary diffuse leukoencephalopathy with spheroids (HDSL; Rademakers et al., 2011). Interestingly, variants of these genes are associated with increased risk for AD and cognitive decline (Efthymiou and Goate, 2017), implying a pivotal role for microglia in AD pathogenesis.

## Genetics of AD

Mutations in the *APP*, *presenilin 1* (*PSEN1*) and *presenilin 2 (PSEN2)* genes can cause Familial AD (FAD), which is typically associated with early-onset (<65 years) AD (Selkoe, 2001; De Strooper and Annaert, 2010; Tcw and Goate, 2017). In contrast, sporadic or LOAD, which accounts for the majority of the cases (90%–95%), results from a complex genetic architecture. *Apolipoprotein E* *epsilon* 4 (*APOE* ε4) is the most common genetic risk factor for AD; APOE4 carriers have a 3–8-fold higher risk of developing AD than non-carriers (Corder et al., 1993). In addition to APOE, recent genome-wide association studies (GWAS) have identified more than 20 additional susceptibility loci associated with AD, including rare coding variants of *TREM2* that confer AD risk comparable to that of *APOE4* carriers (Benitez and Cruchaga, 2013; Guerreiro and Hardy, 2013; Guerreiro et al., 2013). Findings from gene network analyses also revealed higher AD risk associated with several rare variants of genes that are highly expressed in microglia (Dos Santos et al., 2017; Efthymiou and Goate, 2017; Hickman et al., 2018; Ulland and Colonna, 2018), including *complement receptor 1 (CR1*; Corneveaux et al., 2010), the *membrane-spanning 4-domain subfamily A (MS4A)* gene cluster, *ATP-binding cassette transporter A7* (*ABCA7*; Steinberg et al., 2015), the *cluster of differentiation 33* (*CD33*; Bradshaw et al., 2013), *Phospholipase C Gamma 2 (PLCG2)* and *B3 domain-containing transcription factor ABI3* (*ABI3*; Sims et al., 2017). These genetic findings provide strong evidence that microglia-mediated immune response contributes directly to the development of AD.

## Genetic Investigation of TREM2 in AD and Other Neurodegenerative Diseases

The importance of TREM2 in neuronal health was first demonstrated by genetic studies that identified TREM2 variants in families with NHD (also known as polycystic lipomembranous osteodysplasia with sclerosing leukoencephalopathy or PLOSL), a fatal disease characterized by presenile dementia and bone cysts (Paloneva et al., 2001, 2002; Klünemann et al., 2005; Bianchin et al., 2006). Heterozygous rare variants R47H, R62H and H157Y of TREM2 are associated with an increased risk of developing AD in European, African American and Asian populations (Guerreiro et al., 2013; Jonsson et al., 2013; Jin et al., 2014; Jiang et al., 2016). Besides AD, *TREM2* variants have been linked to other neurodegenerative diseases, including ALS (Cady et al., 2014), PD (Rayaprolu et al., 2013) and FTD (Borroni et al., 2014; Lill et al., 2015). A recent study showed that AD mice heterozygous for the *Trem2 R47H* allele were comparable to AD mice lacking one copy of *Trem2* (Cheng-Hathaway et al., 2018). Moreover, in 5XFAD mouse models in which the mouse endogenous *Trem2* was replaced by human normal *TREM2* or human *TREM2 R47H*, Aβ-induced microglial response was found significantly reduced in *TREM2 R47H* mice (Song et al., 2018), further supporting that *R47H* variant impairs TREM2 function *in vivo*. Interestingly, two other independent mouse models with mouse *Trem2* *R47H* knock-in showed reduced *Trem2* mRNA and protein levels, indicating that mouse *Trem2* *R47H* variant activates a cryptic splice site and leads to *Trem2* haploinsufficiency only in mice but not in humans (Xiang et al., 2018). Thus, humanized *TREM2* *R47H* knock-in mice should be used to study the cellular consequences caused by human *TREM2* *R47H* coding variant and it will be interesting to study the genetic variability and the extended mouse phenotype in the context of AD.

TREM2 is highly expressed in white matter in normal human brain, with transcripts detected in all brain regions (Forabosco et al., 2013). It is also highly expressed in plaque-associated microglia in human AD brain (Frank et al., 2008; Yuan et al., 2016; Yin et al., 2017). Studies in animal models have consistently found increased TREM2 expression during aging and disease progression (Guerreiro et al., 2013; Jay et al., 2015; Wang et al., 2015). A growing body of evidence provides new insight into the multifaceted roles of TREM2 in regulating extracellular Aβ pathology (Wang et al., 2016; Lee et al., 2018), hyperphosphorylation and aggregation of tau (Bemiller et al., 2017; Leyns and Holtzman, 2017; Sayed et al., 2018), microgliosis and inflammation in AD (Zhong et al., 2015, 2017b; Zheng et al., 2016; Jay et al., 2017a; Ulrich et al., 2017). Interestingly, it has been reported that TREM2 can be cleaved by the γ-secretase, which is required for the generation of Aβ peptides (Wunderlich et al., 2013). TREM2 has also been found to interact with PSEN1, the key component of the γ-secretase complex, in a manner independent of γ-secretase activity to affect TREM2-mediated phagocytic capacity in microglia (Zhao Y. et al., 2017).

Additionally, the ectodomain of TREM2, namely soluble TREM2 (sTREM2), can be generated by proteolytic processing within the protein stalk (Wunderlich et al., 2013) or by alternative splicing (Jin et al., 2014). sTREM2 has been identified in the CSF of multiple sclerosis (MS) patients (Piccio et al., 2008) and a number of AD cohorts (Gispert et al., 2016; Heslegrave et al., 2016). A recent study found that sTREM2 can trigger microglial activation, induce inflammatory responses and promote microglial survival (Zhong et al., 2017a), which suggests the possibility of targeting sTREM2 as potential AD therapy. These results also provide a paradigm in which sTREM2 may act independently of, and perhaps oppose to, the role of full-length TREM2 in regulating inflammatory responses. Additional work is needed to explore other functions of sTREM2 in microglia and to define the precise molecular pathways downstream of sTREM2 actions.

## Ligands of TREM2 Associated With AD

**Figure 1:**
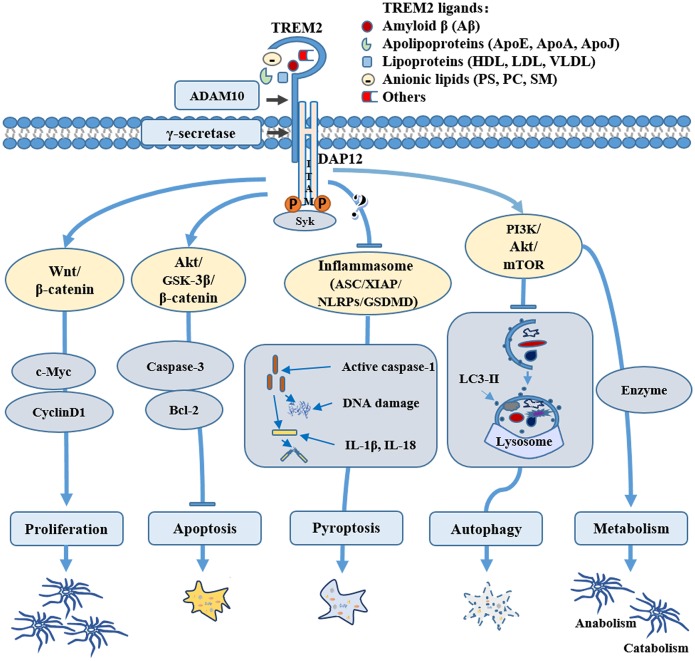
Role of triggering receptor expressed on myeloid cells-2 (TREM2) in microglial survival and metabolism. TREM2 may mediate microglial cell survival through various pathways: TREM2 can promote microglial proliferation by activating the Wnt/β-catenin signaling pathway and inhibit microglial apoptosis through the Akt/GSK3β pathway; TREM2 can regulate the key components of inflammasome, such as NLR family pyrin domain containing 3 (NLRP3) and/or gasdermin D (GSDMD) to inhibit microglial pyroptosis; TREM2 can also activate the PI3K/AKT/mTOR pathway, regulating microglial autophagy and sustaining cellular energetic and biosynthetic metabolism, therefore promoting the survival of microglial cells. Through binding to various ligands, such as amyloid-β (Aβ), apolipoproteins, lipoprotein or anionic lipids, TREM2 can be stimulated physiologically or pathologically, directing microglia to different fates during normal conditions or the pathogenesis of Alzheimer’s disease (AD) and other neurological diseases.

Many potential TREM2 ligands have been proposed (Figure 1), including Aβ, apoE, anionic lipids, etc.

### Aβ

Although the role of TREM2 in regulating Aβ plaques has been controversial, all *Trem2*^−/–^ studies have shown decreased clustering of myeloid or microglial cells around plaques, suggesting a defect in microglia activation (Ulrich et al., 2014; Jay et al., 2015, 2017a; Wang et al., 2015, 2016; Konishi and Kiyama, 2018). Importantly, samples from human R47H carriers also showed fewer microglia clustering around Aβ plaques with diffuse morphology, which are more toxic to neurons (Yuan et al., 2016).

However, how TREM2 impacts microglia-plaque interaction and Aβ-associated cognitive deficits remains unclear. Recent studies have shed new light onto this issue by showing evidence of direct binding of TREM2 to Aβ oligomers with nanomolar affinity, whereas AD-associated TREM2 mutations show reduced Aβ binding (Zhao et al., 2018; Zhong et al., 2018). Very recently, TREM2 was found to interact directly with various forms of Aβ, with the highest affinity interactions with soluble Aβ42 oligomers (Lessard et al., 2018). These findings have offered key insight into the role of TREM2 in Aβ pathologies, in which TREM2 strongly affects the ability of microglia to respond to plaques (Colonna and Wang, 2016; Udeochu et al., 2018). Indeed, increasing TREM2 gene dosage reduces plaque area and appears to promote the formation of plaques that are more compact and associated with fewer dystrophic neurites (Lee et al., 2018).

### ApoE

In 2015, two groups independently identified a direct interaction between TREM2 and apoE (Atagi et al., 2015; Bailey et al., 2015). Both groups found that TREM2 binds all apoE isoforms, lipidated and non-lipidated, and that the R47H variant reduced its binding affinity to apoE. Separate groups identified a set of lipoprotein particles, including very low density lipoprotein (VLDL), LDL and high-density lipoprotein (HDL), as well as apolipoproteins, including CLU/apoJ and apoE, as TREM2 ligands (Yeh et al., 2016; Jay et al., 2017b; Song et al., 2017). Binding of these ligands was abolished or reduced in disease-associated TREM2 mutations (Song et al., 2017).

Given that apoE genotype is by far the strongest genetic factor modulating Aβ deposition, and the risk of suffering AD associated with TREM2 variants is comparable to that of the apoE4 allele, an interaction between apoE and TREM2 connects these two key AD risk factors, which could have pivotal roles in the pathogenesis of AD (Engstrom et al., 2017; Jendresen et al., 2017; Pankiewicz et al., 2017). Indeed, the TREM2-apoE pathway has been identified as a major regulator of microglia phenotypic change in neurodegenerative diseases (Krasemann et al., 2017). TREM2 activation of the apoE signaling pathway can restore the homeostatic signature of microglia in ALS and AD mouse models, and prevent neuronal loss in an acute model of neurodegeneration (Krasemann et al., 2017). Interestingly, recent work demonstrated that apoE is required for microglial association to Aβ plaques where it performs a plaque-trimming function similar to that of TREM2; thus supporting an apoE-TREM2 axis in mediating microglial function (Ulrich et al., 2018). These studies indicate that apoE and TREM2 may share similar mechanisms in regulating microglial cell responses in Aβ pathology (Shi and Holtzman, 2018).

### Anionic Lipids and Other Ligands

Bacteria and polyanionic molecules, including dextran sulfate, lipopolysaccharide (LPS) and lipoteichoic acid (LTA), were the first potential ligands identified for TREM2 (Daws et al., 2003; Charles et al., 2008; Quan et al., 2008; N’Diaye et al., 2009). TREM2 has also been shown to bind a number of anionic molecules from mammalian cells. Studies using solid-state ELISA or TREM2 reporter cells support the concept of phospholipids as TREM2 ligands, including phosphatidylethanolamine (PE), phosphatidylserine (PS), and cardiolipin, phosphatidylcholine (PC) or sphingomyelin (SM; Cannon et al., 2012; Poliani et al., 2015; Wang et al., 2015). In addition to phospholipids, nucleic acids released by damaged cells can also act as TREM2 ligands. Both cellular fractions containing nuclei and purified DNA can activate TREM2 signaling (Kawabori et al., 2015). Furthermore, it has been shown that TREM2 can also bind proteoglycans (such as heparan sulfates or HS), which are a large group of cell-surface proteins. Interestingly, proteoglycan binding to TREM2 is impacted by AD risk variants (Kober et al., 2016). The potential roles of these ligands in TREM2 signaling pathway in physiological and pathological conditions is currently the target of intense research as they may represent potential new sources of therapeutic and diagnostic tools.

## TREM2 and Microglial Survival

TREM2-mediated microglia activation can display a number of forms (Figure 1), including promotion of cell survival and proliferation, regulation of cytokine production, and/or phagocytosis (Schafer and Stevens, 2015; Leyns and Holtzman, 2017). TREM2 stimulation by these ligands is also important for microglial function in response to pathogens and other causes of cell damage. For example, Aβ-induced microglial depolarization, K^+^ inward current induction, cytokine expression and secretion, migration, proliferation, apoptosis and morphological changes are all dependent on TREM2 activation (Zhao et al., 2018). Moreover, recent evidence revealed the crucial role of TREM2 in maintaining microglial cell survival (Wang et al., 2015) and optimal metabolic fitness (Ulland et al., 2017).

### TREM2 and Microglial Proliferation

Studies have found that mice lacking TREM2 (*Trem2*^−/–^) have fewer microglia and increased microglia apoptosis (Ulland et al., 2015; Jay et al., 2017b; Yeh et al., 2017). Cell proliferation was also reduced in microglia derived from *Trem2*^−/–^ mice compared with those from wild type (WT) mice both *in vivo* and *in vitro* (Zheng et al., 2017). In *Trem2*^−/–^ mouse brain, TREM2 deficiency reduced the viability and proliferation of microglia, reduced microgliosis, induced cell cycle arrest at the G1/S checkpoint, and decreased the stability of β-catenin, a key component of the canonical Wnt signaling pathway responsible for maintaining key biological processes, including cell survival (Zheng et al., 2017). TREM2 is also involved in macrophage CSF (M-CSF) mediated survival and proliferation of macrophages and osteoclast precursors. M-CSF, also known as CSF-1, acting via the CSF-1R can induce calmodulin-dependent kinase-mediated phosphorylation of β-catenin in a TREM2-dependent mechanism (Otero et al., 2009, 2012). However, whether there is a synergy between the TREM2 and CSF1R remains elusive and further studies on the cell- and tissue-specific functions of the TREM2/CSF1R signaling pathway in healthy and diseased brains may provide insight into this (Kober and Brett, 2017).

### TREM2 and Microglial Apoptosis

TREM2 deficient microglia show high rates of apoptotic cell death (Wang et al., 2015). Our recent work also demonstrated that TREM2 depletion accelerates microglial apoptosis by showing significantly increased levels of cleaved caspase 3 and decreased levels of pro-caspase 3 and Bcl-2 in *Trem2*^−/–^ microglia compared to WT microglia (Zheng et al., 2017). Furthermore, sTREM2, purified from conditioned media of transfected HEK293T cells, was found to attenuate apoptotic cell death caused by withdrawal of granulocyte-M-CSF (GM-CSF) in primary microglia cultures from both WT and *Trem2*^−/–^ mice (Zhong et al., 2017a). Indeed, previous studies have shown that TREM2 suppresses apoptosis in lung macrophages during Sendai virus infection (Wu et al., 2015). Together these results imply a vital role for TREM2-mediated signaling in programmed cell death.

### TREM2 and Microglial Autophagy

The term autophagy (derived from the Greek words auto: self and phagos: eating) indicates a self-sacrificing mechanism of cell elimination and removal (Eisenberg-Lerner et al., 2009; Zhu and Zhang, 2018). At the basal level, autophagy plays a vital role in keeping cell homeostasis through digestion of dysfunctional organelles and proteins (Metaxakis et al., 2018; Mizushima, 2018). Defective autophagy pathways or alterations in autophagy-related genes have been revealed in various human pathologies including neurodegenerative disorders and microglia dysfunction (Mizushima et al., 2008; Jiang and Mizushima, 2014; Kim et al., 2016; Moors et al., 2017). Autophagy involves the activation of three major autophagic pathways: macroautophagy, microautophagy and chaperone-mediated autophagy (CMA); and the final destination of all three autophagic pathways is the lysosome (Ghavami et al., 2014; Deng et al., 2017).

Studies have provided evidence on how autophagy may affect microglial function during progression of age-associated neurodegenerative diseases (Plaza-Zabala et al., 2017). Recycling of TREM2 in microglia is regulated by Beclin-1, a protein involved in autophagy (Salminen et al., 2013) and levels of TREM2 are reduced in AD brain microglia (Lucin et al., 2013), implying a potential link between TREM2 and autophagy. A recent study by Colonna and Wang (2016) provided evidence in support of this prediction by showing deficient TREM2-mediated autophagy in human AD brain and mouse AD model (Ulland et al., 2017). The lipidation of microtubule-associated light chain 3 (LC3) is an important marker and effector of autophagy. The ratio of lipidated LC3II to non-lipidated LC3I was markedly higher in microglia from 5XFAD mice lacking TREM2 (*Trem2*^−/–^) than in WT 5XFAD mice. This is consistent with the increased number of autophagic vesicles observed in TREM2-deficient microglia (Ulland et al., 2017). These results indicate that TREM2 plays an important role in microglial autophagy; however, identification of the mechanism and/or signaling pathways need to be verified.

### TREM2 and Microglial Pyroptosis

Inflammasome is a type of tissue-damage sensor that is necessary for the conversion of IL-1β immature pro-form to the mature, active form and it is implicated in a form of cell death called pyroptosis (Keller et al., 2008; Latz et al., 2013; Sharma and Kanneganti, 2016). Increasing evidence shows aberrant expression of inflammasome-related proteins in AD brain (Tan et al., 2014; Olsen and Singhrao, 2016; Saresella et al., 2016). Although one study demonstrated that TREM2 promoted host resistance against *P. aeruginosa* by inhibiting caspase-1-dependent pyroptosis (Qu et al., 2018), it is still unclear whether and how TREM2 may be involved in inflammasomes and pyroptosis in microglia. Due to the complex relationship between TREM2 and inflammatory pathways (Jay et al., 2017b), further research is needed to elucidate whether and how TREM2 plays a biological role in microglial pyroptosis in AD (Figure 1). For example, it would be interesting to investigate whether TREM2 can regulate key components of inflammasome, such as NLR family pyrin domain containing 3 (NLRP3) and/or gasdermin D (GSDMD) in AD.

## TREM2 and Metabolism

Similar to other cell types, microglia express the full complement of gene products required for both glycolytic and oxidative metabolism (Ma et al., 2017). Evidence suggests that microglia metabolize glucose, acetoacetate (AcAc) and β-hydroxybutyrate (BHB), increase aerobic glycolysis and decrease respiration when activated by various stimuli (Ghosh et al., 2018; Zhou et al., 2018). Mutations in TREM2 were found to disrupt microglial energy state and function, thus sabotaging microglia’s ability to protect the brain against toxic amyloid plaques (Hong and Stevens, 2017; Ulland et al., 2017). Ulland et al. (2017) also found that microglia in AD patients carrying TREM2 risk variants and in TREM2-deficient mice with AD-like pathology were defective in rapamycin (mTOR) signaling, which affects ATP levels and biosynthetic pathways. Metabolic derailment and autophagy were offset *in vitro* through Dectin-1, a receptor that elicits TREM2-like intracellular signals, and cyclocreatine, a creatine analog that acts as a source of ATP (Ulland et al., 2017). This study indicates that TREM2 enables microglial responses during AD by sustaining cellular energetic and biosynthetic metabolism. TREM2 has also been linked to lipid metabolism. TREM2 promotes adipogenesis and diet-induced obesity by upregulating adipogenic regulators in association with inhibiting the Wnt10b/β-catenin signaling pathway (Park et al., 2015). The connection between TREM2 and lipid metabolic pathways has also been confirmed by expression analysis (Piccio et al., 2008; Poliani et al., 2015). Studies consistently demonstrate that a TREM2 loss-of-function mutation T66M can cause a significant reduction in brain glucose metabolism (Kleinberger et al., 2017). These studies indicate that TREM2 play critical roles in maintaining the anabolism and catabolism of nutrients.

## Conclusion and Prospects

Substantial genetic findings point to a central role of microglia in neurodegenerative diseases. TREM2 can support microglial cell survival by promoting microglial proliferation and inhibiting apoptosis, autophagy and perhaps pyroptosis. TREM2 also supports a higher metabolic rate in microglia, while dysfunction of TREM2 impairs brain metabolism. Besides anionic lipids, several new ligands of TREM2, such as Aβ and apoE, have been identified. It remains unclear what exact signaling pathways these ligands activate and how they are involved in microglia survival and metabolism. Therefore, further research is needed to elucidate the biological role of these ligands in TREM2 signaling pathway and in microglia survival in AD pathogenesis. Given that clinical trials targeting the accumulation of Aβ and hyperphosphorylated tau in the brain have nearly all ended in failure, deciphering the role of TREM2 in microglia will advance our understanding and provide novel therapeutic strategies for the treatment of AD and other neurodegenerative diseases.

## Author Contributions

HZ wrote and reviewed the manuscript. BC drew the figures. XL arranged the references. YL, XC and YZ reviewed the manuscript.

## Conflict of Interest Statement

The authors declare that the research was conducted in the absence of any commercial or financial relationships that could be construed as a potential conflict of interest.
